# Healthy, Active Aging for People and Dogs

**DOI:** 10.3389/fvets.2021.655191

**Published:** 2021-06-07

**Authors:** Sandra McCune, Daniel Promislow

**Affiliations:** ^1^School of Psychology, School of Life Sciences, University of Lincoln, Lincoln, United Kingdom; ^2^Animal Matters Consultancy Ltd., Stamford, United Kingdom; ^3^Department of Lab Medicine and Pathology, University of Washington School of Medicine, Seattle, WA, United States; ^4^Department of Biology, University of Washington, Seattle, WA, United States

**Keywords:** aging, dogs, human-animal interaction, healthspan, healthy aging

## Abstract

Dogs act as companions who provide us with emotional and physical support. Their shorter lifespans compel us to learn about the challenges and gifts of caring for older individuals. Our companion dogs can be exemplars of healthy or unhealthy aging, and sentinels of environmental factors that might increase or decrease our own healthy lifespan. In recent years, the field of aging has emphasized not just lifespan, but healthspan—the period of healthy, active lifespan. This focus on healthy, active aging is reflected in the World Health Organization's current focus on healthy aging for the next decade and the 2016 Healthy Aging in Action initiative in the US. This paper explores the current research into aging in both people and companion dogs, and in particular, how the relationship between older adults and dogs impacts healthy, active aging for both parties. The human-dog relationship faces many challenges as dogs, and people, age. We discuss potential solutions to these challenges, including suggestions for ways to continue contact with dogs if dog ownership is no longer possible for an older person. Future research directions are outlined in order to encourage the building of a stronger evidence base for the role of dogs in the lives of older adults.

## Introduction

Humans and our non-human animal companions share many attributes, and perhaps none more so than the experience of aging. As we and our pets grow older, we experience a steady physiological decline, leading to age-specific increases in the risks of morbidity and mortality. Even in our youth, we observe this in our grandparents and parents, and often quite dramatically in our pets, and eventually, in our own lives.

Aging is a powerful phenomenon. In human populations, age is the single greatest risk factor for most of the common causes of mortality (1), often by orders of magnitude compared to the next most important risk factors, and aging also has tremendous psychological, social and economic impacts (2). We can think of aging as a broad, unifying principle—a conceptual bridge linking diverse ways in which we understand the world, from molecular and evolutionary biology, to demography and economics, to history and the fine arts, and more. It also provides a unique link between our pets and ourselves. Given the rapidity with which our pets age relative to us, they are a powerful reminder of what lies ahead for us, and they provide us with an opportunity to learn how to age well.

The past 30 years of aging research has led to tremendous advances in our understanding of the basic biology of aging, helping us to better understand aging both in our own species and our companion dogs. In this review we start with a brief discussion on the impact of aging in human populations (about which we know a great deal), about aging in dogs (about which we know much less), and the similarities and differences between the two. We then turn to an exploration of how the human experience of aging impacts our relationship with dogs, and how, in turn, aging in dogs affects human-animal interaction. Finally, we consider some of the important questions that arise from a consideration of the mutual experience of aging in humans and dogs.

### Similarities and Differences in the Biology of Aging

**Figure 1:**
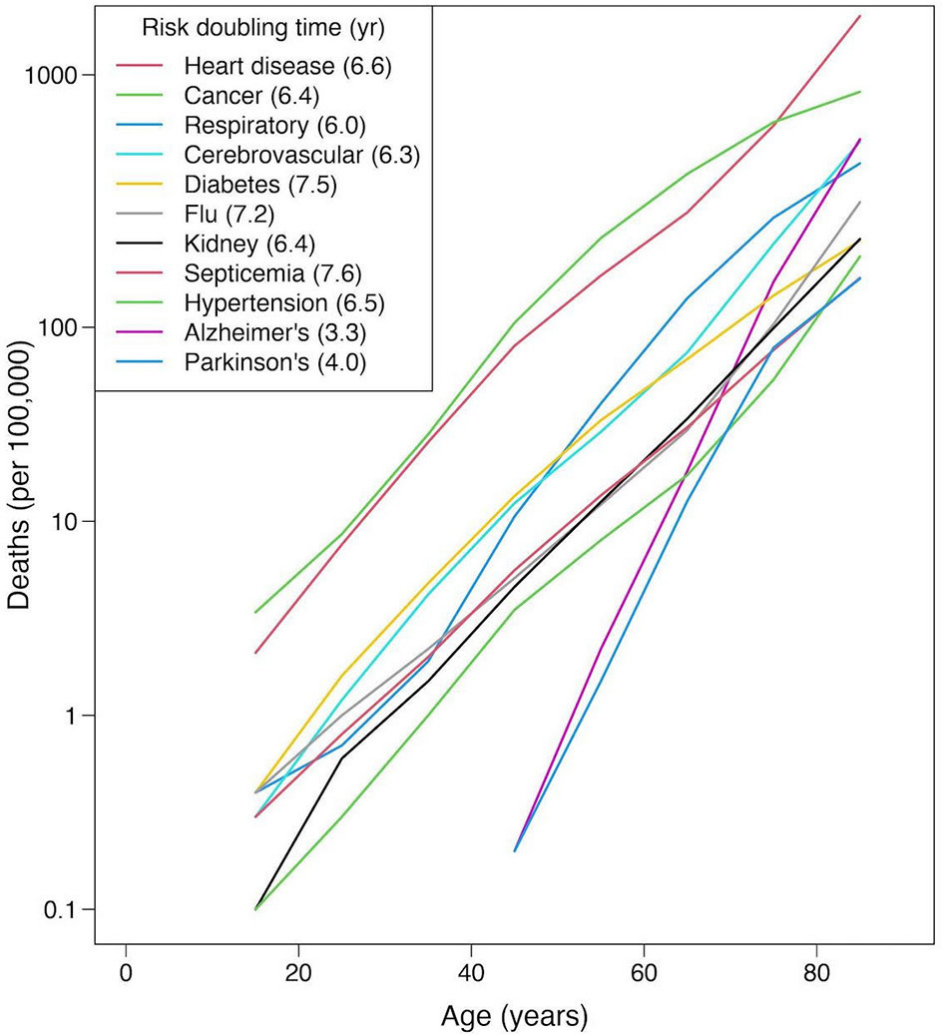
Annual risk for diverse causes of mortality, based on 2013 US Centers for Disease Control National Vital Statistics Report. Note for virtually every major cause of mortality, risk increases exponentially with age (a straight line on a log-linear plot). The inset legend includes the mortality rate doubling time-the number of years it takes to increase mortality risk m to 2 m (4).

What do we mean by aging? There are numerous ways to define just what we mean by aging (also commonly known as senescence). Here, we define aging as the intrinsic physiological decline that occurs as organisms age, leading to a decline in fertility and fecundity, and an age-related increase in the risk of morbidity and mortality (3). As we mentioned above, age is the single greatest risk factor for most major causes of mortality in adults. This is shown in Figure 1, which illustrates that the age-specific increase in risk is exponential (linear on a logarithmic scale) for the major causes of human mortality in the United States.

While not all species suffer from the same diseases, everyone eventually dies. The way that age affects the risk of death shows striking similarities across almost all animal species (5–7). The risk of dying is typically high immediately after birth, declines to a minimum in early adulthood, and then begins to climb steadily, and like the pattern of individual diseases, increases exponentially. In humans, after age 30 the risk of death doubles about every 7–8 years (8). The shape of this relationship is notably similar in dogs (9). But there are important differences in the causes of this mortality. Some diseases, like cancer, are relatively common causes of mortality in both species, and increase with age not only in humans (Figure 1) but also in dogs (10–12). In contrast, while cardiovascular disease rises steadily to become the single greatest cause of mortality in humans (Figure 1), in dogs it appears to be rare, and independent of age (13, 14).

It is clear from these prior studies that both humans and dogs age, but *why* do species age? In the 1950's, Peter Medawar (15) and George Williams (16) laid out the evolutionary theory that explains why aging is inevitable. In short, if a germ-line mutation reduces fitness early in life, selection will tend to eliminate that mutation. But if that same effect is limited to later in life, selection will be relatively ineffective at weeding out the mutation. Over evolutionary time, these late-acting deleterious mutations will accumulate so populations will tend to carry large numbers of these variants, leading to senescent decline. And indeed, in almost every species examined, in the lab and in the wild, we see signs of aging (6, 17).

Interestingly, not only are the demographic patterns of aging conserved. The pathophysiological consequences of aging are also strikingly similar throughout the animal kingdom, from shortened telomeres, to mitochondrial dysfunction, to loss of the ability to maintain protein structure, and more (18). Turning from the consequences of aging to the molecular causes, over the past 30 years, researchers have identified a number of pathways which, when altered through drugs, diet, or genetic engineering, increase lifespan and healthspan in diverse lab organisms, including yeast, nematode worms, fruit flies and mice (19, 20).

These are exciting discoveries, but it remains to be seen whether these molecular pathways discovered in the lab can also explain variation in aging and age-related traits that we observe in the real world. In populations outside of the lab, the differences we observe among individuals—height, shape, behavior, age-specific risk of disease—are due to differences in genotype, and in the environments that they experience from the moment of conception and throughout their life. In fact, quantitative genetics teaches us that within a population, all of the variation that we observe for any of these traits, or *phenotypes* (**P**), is due to the sum of the genetic (**G**) and environmental (**E**) variation, and the interaction between the two: **P** = **G** + **E** + cov(**G, E**).

To tease apart the complex network of mechanisms by which **G** and **E** ultimately shape aging-related traits, we have been led to consider companion dogs. We are motivated to think about aging in dogs not simply because of their physical proximity to us, but also because they show the most variability of any mammal, not only for morphology and behavior (21), but also for patterns of morbidity and mortality (13). From the smallest to largest breeds, size differs by more than 50-fold (22), and this dramatic size variation is closely related to lifespan, with large-breed dogs typically much shorter-lived than small-breed dogs (23). Given that dogs and humans both have considerable genetic variation, many similarities in disease, and a shared environment, with their much shorter lifespan, dogs provide us with an excellent opportunity to transfer lab discoveries to the “real world.” And given the much shorter lifespan in dogs, we might be able to more easily tease apart how genes and environment shape aging in dogs than in humans.

We are just at the beginning of this work whose goal is to uncover the causes and consequences of aging in dogs (24, 25). These studies are made possible not only by the nature of how dogs age, but also by the close relationship dogs have with their owners, who generously share these data with the researchers. These studies are just one small example of the incredibly rich nature of human-animal interaction, and the relationships it can lead to, in which aging is a central component. This leads to many fascinating questions about the ways that aging in both dogs and humans shaped the nature of this complex bond. In the following sections, we explore this in detail.

### Aging and Human-Animal Interaction

Sharing our lives with pets is a global phenomenon and has been a central feature of human society for thousands of years (26). An increasing body of evidence indicates that pets provide us with physical and emotional benefits (27–29). They encourage us to be more active, make us laugh, provide comfort and affection, help us feel safer, and can even help us connect with our neighbors and make new friends. This companionship may be especially important for older adults as their social networks shrink. As we describe above, for many of us, our companion dogs provide us with our first direct and intimate experience of aging. Even as children, many of us first experience the challenges and gifts of caring for an older individual, and of navigating the emotionally and psychologically difficult terrain around end-of-life care through pet ownership [e.g., (30)].

Since older humans and dogs are both subject to psychological and physical changes as they age, it is important to find creative ways to address the health needs of both these populations. As we develop ever more sophisticated ways to define healthy or unhealthy aging in our companion dogs, what we learn is likely to translate to humans as well. For example, in looking for environmental determinants of healthy aging, given the relatively short lifespan of dogs, we might observe the impact of both beneficial and detrimental environmental factors on aging in dogs long before similar impacts would appear in humans. In this sense, dogs could serve as sentinels of environmental factors that might increase or decrease our own healthy lifespan (31).

### Causes and Consequences of Aging

How can we understand the enormous variation in patterns of aging? Research on human aging has sought first to measure variation in lifespan, and then to measure the degree to which this variation is explained by genes and environment. It turns out that about 20–30% of the variation in human lifespan is due to genetic factors [(32, 33), but see (34)]. So it pays to have long-lived grandparents, but environmental factors make a big difference. The next step has been to identify which genes, and what environmental factors, contribute most to lifespan and age-related traits. In humans, one gene in particular, *ApoE*, has been shown to be associated with lifespan in studies of many different populations. There are particular alleles of this gene that are associated both with high risk of Alzheimer's Disease and short lifespan. Notably few other genes have been found in these studies.

There are considerable challenges in finding the many genetic and environmental factors that contribute to variation in aging. In particular, many genes that affect aging have extremely small effects, and so are hard to identify in even the largest studies (35). But humans are also hard to study because they live for so long. We have learned a great deal from numerous long-term longitudinal studies of human aging [e.g., (36–38)], but because we live so long, these studies take a long time—certainly longer than the duration of a graduate student or post-doc, and even the career of a scientist, interested in aging studies.

With this in mind, researchers have turned to the dog as a powerful model system to study aging in a natural population. As we mentioned above, dogs share similar mortality trajectories as humans, and similar pathophysiological processes (13, 14). Moreover, they share our environment, and so are likely to have at least some environmental risk factors in common with humans.

The first large-scale studies on dog aging have relied on retrospective analysis of existing data. These studies have provided overviews of the variation in lifespan among breeds, and the effect of size, age, and inbreeding on risk of disease and death (13, 14, 39–43). Researchers have also sought to identify genes associated with lifespan in dogs (44, 45). Given the close correlation between size and lifespan, it is particularly challenging to disentangle the two. For example, size in dogs is influenced by many genes, with one particular gene, Insulin-like Growth Factor 1 (IGF1), playing a major role. Large- and giant-breed dogs tend to carry two copies of the ancestral (wolf-like) IGF1 allele, while toy breeds tend to have two copies of a derived allele. Notably, IGF1 is also associated with lifespan in laboratory studies (46) and even in some human populations (47, 48). This has led some to suggest that large-breed dogs are short lived because of the IGF1 allele they carry. While the frequency of the large-size IGF1 allele is associated with shorter mean lifespan across breeds, we do not yet know if there is a causal relationship.

To fully understand how genes and environment shape patterns of aging, the gold standard is the long-term longitudinal study. Human cohort studies around the world have taught us much about genetic and environmental risk factors for a whole range of diseases [e.g., (36–38)]. Among the very many lessons learned, these longitudinal studies have taught us that smoking increases the risk of stroke (49), that a diet rich in fruits and vegetables promotes healthy aging (50), and that early-life socioeconomic status impacts late-life health (51). Dozens of cohort studies support the benefits of both non-vigorous and vigorous exercise in reducing overall mortality risk (52, 53). This latter finding has led many to consider that the potential benefits of dog ownership for older people may be due to increased frequency and/or duration of exercise. We explore this further below.

Inspired by what we know about variation in aging in dogs, and by the success of human cohort studies, more recently, researchers have initiated large-scale longitudinal studies, setting out to follow thousands of companion dogs throughout their lives. The Golden Retriever Lifetime Study (GRLS) (24) is designed to better understanding the underlying causal mechanisms for variation in cancer in that breed, and the Dog Aging Project (DAP) (25) has set out to understand the genetic and environmental determinants of healthy aging by studying all breeds. These and several other ongoing large-scale studies in dogs (54–57) benefit from the power of community science, where dog owners in the general community generously share data about their dogs with researchers, with potential benefits to people, to dogs, and to science. Despite the sophisticated health care system to which many dogs have access, we know relatively little about what healthy aging looks like in dogs. As with human medicine, there are many veterinary specialties, but geriatrics is not among them. Projects like GRLS and DAP will provide the data needed to better understand what healthy aging looks like in dogs, what factors are most likely to promote healthy aging, and whether these findings can be translated to human populations.

This paper explores the current research into aging in both people and companion dogs, and in particular, how the relationship between older adults and dogs impacts healthy, active aging for both parties. The human-dog relationship faces many challenges as dogs and people age. In recent years, the field of aging has emphasized not just lifespan, but healthspan—the period of healthy, active lifespan. This focus on healthy, active aging is reflected in the World Health Organization's (WHO) initiative for a decade of focus on healthy aging (58) and recent initiatives from the US Surgeon General on Healthy Aging in Action (59). As owners age, dogs may be particularly important for maintaining social connection with others, heart health, mobility and even cognitive function. However, older adults may reach a stage of decline when they can no longer adequately care for their dog. Aging dogs may place additional demands and costs on their owner.

### Healthy Active Human Aging

Life expectancy is increasing in many parts of the world and with that come new opportunities but also unprecedented challenges. Healthy aging provides the opportunity for older people to take an active part in society and enjoy an independent and high quality of life for longer. The WHO defines *healthy aging* “as the process of developing and maintaining the functional ability that enables wellbeing in older age” and it defines *functional ability* as being “about having the capabilities that enable all people to be and do what they have reason to value” (60). Given the rapidly growing number of older adults in the coming decades (61), innovative approaches to promote healthy aging are increasingly important. Extending the period of independent living in older adults can have a positive impact on quality of life and healthcare costs.

## The role of pets in healthy active human aging

Our world is changing, and as populations become increasingly aged in many countries, communities need to be better able to support this societal shift. New, innovative approaches to help older adults remain healthier for longer as they age may extend their healthspan but also potentially reduce the burden of healthcare costs (62). The role that pets play in creating healthier, more engaged communities should not be overlooked. There is an increasing body of evidence suggesting that pets may offer a range of health benefits supporting older adults to retain their physical and mental health, independence, social connectedness and engagement (63–66). Pets also offer humans opportunities to nurture and feel needed, to provide a purpose, structure and routine for daily life (67), to enhance feelings of security (68), to give and receive affection, and to maintain older adults' ability to care for themselves independently (69).

Beyond physical benefits, pets may help us meet our basic need to connect with “another.” Pets can provide a reason to get out of bed in the morning, a partner for walks through the neighborhood, and a positive topic of conversation with friends and neighbors. Opportunities to provide nurturance to others and to give and receive affection may decrease as we age, but pets are constant companions who can make us feel needed, valued, and loved. Several studies have shown that pets can often fulfill the four roles of an attachment figure proposed by Ainsworth (70). Specifically, many pet owners report that their pets are enjoyable and comforting (71), missed when absent (72), and sought out in times of distress (73).

### Physical Health and Mobility

Exercising in later life can be a challenge despite its well-known benefits (59). In the U.K., only one in four people aged between 65 and 74 exercise regularly (74). By age 75, about one in three men and one in two women in the US engage in no physical activity (https://www.cdc.gov/physicalactivity). Adults aged over 50 years who frequently walked their dog were more likely to report having a sense of community, more likely to achieve the recommended levels of physical activity (at least 150 min/week), and less likely to be sedentary than those who did not live with a dog (75–80). In one study, they achieved an average of 22 additional minutes of walking per day (~2,760 steps), compared to non-dog owners (74). Results from the Health & Retirement Study's longitudinal survey indicated that dog walking was associated with more frequent moderate and vigorous exercise, lower body mass index, fewer limitations in activities of daily living and fewer doctor visits (81). Dog owners walked faster and were more likely to maintain their walking speed over a 3-year study than dog owners who did not walk their dog or non-owners (82). Walking speed is thought to be an indicator of balance, and for older adults, balance is crucial for preventing falls and maintaining independence (83).

The American Heart Association has issued a statement in support of the role that dog ownership can play in reducing the risk of developing cardiovascular disease (CVD) (84): “Pet ownership, particularly dog ownership, is probably associated with decreased cardiovascular disease” and “…may have some causal role in reducing CVD risk.” One landmark study determined that risk of death from cardiovascular disease was decreased by 26% for pet owners compared to non-pet owners (85) following a serious heart attack, a result that was later replicated in larger cohort studies (86, 87). Presence of pet was associated with lower blood pressure in older adult patients with hypertension (88).

### Socio-Emotional and Cognitive Health

The increased risk of isolation and loneliness in older adults has a profound impact on health and well-being, and is often associated with depression (89, 90), lower overall life satisfaction (91), and with reductions in mobility and activities of daily living (91, 92). The effect of loneliness and isolation on mortality is comparable to the impact of well-known risk factors such as obesity and smoking cigarettes (93). In a meta-analysis of 70 studies, the likelihood of death was 26% higher for those reporting loneliness, 29% higher for those experiencing social isolation, and 32% higher for those living alone (94). Pets can make us feel needed and valued. Older adults having contact with dogs reported reduced levels of loneliness and improved mental functioning (95), although some studies of loneliness showed little effect of interaction with dogs. It may be that some pet owners are lonely people who get a dog to alleviate loneliness while for others, dogs may be a protective factor against loneliness developing. Pet ownership is not a homogenous experience, which may explain the mixed results in studies of the impact of dogs on loneliness. Randomized controlled trials are needed to more definitively establish the relationship between pet ownership and loneliness (66).

Other socio-emotional benefits of human-dog interaction reported include older adults with dementia showing significant decreases in agitated behavior and increases in social interaction when a pet visited (96, 97). Pet ownership was associated with less depression following spousal bereavement (98).

A recent systematic review evaluated 145 research studies on the topics of human-animal interaction and physical health and exercise, depression and anxiety, and loneliness and social functioning in older adults (66). Among the less robust studies reviewed, pet attachment was associated with reduced loneliness in older adults, mediated the relationship between loneliness and health, and was viewed as a coping resource for loneliness. In contrast, most of the higher quality studies indicated no positive effect of pet ownership. However, one study found that individuals over 60 who lived alone reported their pets as particularly effective in attenuating loneliness and another found that higher levels of pet attachment related to less loneliness.

### Pets as Social Capital

Social Capital is a concept that captures trust between people (including those we do not know personally), networks of social support, the exchange of favors with neighbors and civic engagement. Many studies show that Social Capital is positively associated with important social indicators including mental health, education, crime deterrence and community safety. Pet Ownership, particularly dog ownership, is linked to higher levels of social capital and civic engagement (99). In a larger study, over 2,500 pet owners and non-pet owners were surveyed across four cities (Perth in Australia and San Diego, Portland and Nashville in the US) reasonably comparable in size, urban density and climate (100). In all four cities, pet ownership was significantly associated with higher social capital compared with not owning a pet. This held true after adjusting for a raft of demographic factors that might influence people's connections in their neighborhood. Among Pet Owners of all types, social capital was highest in dog owners who walked their dog. Dog owners were twice as likely as non-pet owners to have gotten to know someone in their neighborhood (101).

Approximately 40% of pet owners reported receiving social support from people they met through their pet (101). Impact of pets goes beyond individuals. Emerging evidence indicates that pets may act as a social bridge between people and contribute to “ties that bind” societies and communities together, contributing to a civil society and healthy lifestyle. Pet owners were also more likely to be concerned about and active in their communities (101).

Not everyone wants a pet or indeed probably should have a pet. But given pet ownership is common, it should not be overlooked as a means of potentially strengthening communities. It follows that cities and neighborhoods should be “pet-friendly” to encourage responsible pet ownership. “No Pet” clauses in rental or social housing have been a strong barrier for pet ownership but recently we are seeing a change in some countries. For example, the UK government's Model Tenancy Agreement (102) includes provision for pets.

## Healthy active canine aging

The burden of caring for an elderly pet can be particularly challenging for an elderly owner. Thus, maximizing healthy aging in dogs can improve quality of life not just for the dog, but also for the aging owner. And the goal is not simply to maximize longevity, which could lead to extended periods of poor-quality life with high morbidity, but rather to maximize the period of healthy lifespan, or “healthspan” (103, 104). In recent years, this same notion has been introduced into the canine literature (14, 25, 105).

As any dog owner knows, like humans, dogs slow down as they age. With age comes not only decreased mobility (106–108), but also changes in diverse behaviors (109, 110), age-related loss of cognitive function that, in many ways, mirror those in humans (57, 111), declines in physiological function (112) and increases in the risk of morbidity (13) and overall mortality (9, 42, 43).

Decades of epidemiological studies have shown us what we can do to maximize our own healthspan—eat healthy foods and in moderation, do not smoke, get plenty of exercise. And as we mentioned above, having long-lived parents and grandparents helps too. What about dogs? We know that genetics plays a major role in determining life expectancy in dogs, with small breeds typically living considerably longer than most large breeds (113). But we know surprisingly little about the effects of diet, exercise or other environmental factors on canine healthspan.

We do know a considerable amount about diet and health in dogs, though there are few carefully designed clinical studies focused on diet and age-related disease or longevity [e.g., (114)]. Inspired by decades of studies in laboratory organisms showing that dietary restriction can enhance healthy lifespan (115, 116), a long-term study of diet restriction in a colony of Golden Retrievers suggested that the same might be true in dogs (117, 118), though the effects in this study appear to be due primarily to increased adverse effects of osteoarthritis in *ad libitum* fed dogs.

The literature on how diet might maximize healthy aging in dogs is permeated with anecdotal claims and is in need of large-scale long-term studies. Where we see more consistency is in the literature on the risks of obesity in dogs (119). Obesity is most notably associated with increased problems with osteoarthritis in dogs, but also can affect overall quality of life (120). But here, too, more studies are needed to better understand if and how obesity might impact other health risks in dogs (121). Moreover, while we know that exercise is associated with decreased risk of obesity [e.g., (122)], to our knowledge no one has yet shown that exercise in dogs is associated with increased healthspan.

Given the impact of aging in the lives of both dogs and their owners, it is notable that while veterinary medicine, like human medicine, has a broad set of specialties, such as cardiology, oncology, parasitology, nutrition, and so forth, notably missing from the list is geriatrics. Dog owners and veterinarians alike can recognize an older dog, and the infirmities that come with age. But the in-depth knowledge of aging that a human geriatrician possesses does not yet exist in the veterinary medical landscape. With studies like the Dog Aging Project (25) and the Golden Retriever Lifetime Study (24) now underway, data from these studies should reveal the factors that influence risks of aging and age-related disease, the diagnostic parameters and particular treatments that might be most appropriate for older dogs, and finally, ways to increase healthspan in dogs.

## Advancing research on older adults and pets

As we described above, we are now seeing well-funded, large-scale studies of aging in dogs (24, 25, 54–57). These studies complement the many ongoing studies in human populations. At the same time, the call continues for high quality, well-designed research on the special bond between older adults and pets as a means of improving human health in a rapidly aging world. Research questions need to move beyond pet ownership as a binary question and should be framed to examine different variables including: the extent and quality of regular interaction with dogs; pet keeping history over a person's lifetime; the length of time they have had a relationship with their current dog; contact with other people's dogs or through animal-assisted interventions; and importantly, the relationship between a person's desire for, and the reality of, pet interaction as a factor in observed human health outcomes (123). Priorities include how human-animal interaction (HAI) impacts on major transitions or events in the lives of older adults such as retirement, the death of a spouse, and seeking or moving from independent living into sheltered accommodation or an institutionalized facility (67, 124). Recent systematic reviews within sub-populations are welcome but more research is needed on more ethnically and culturally diverse populations (125).

High quality, well-designed research requires standardized measures that are well-validated so that different research studies can be compared; well-designed research with comparison groups and adequate sample sizes; a focus on specific outcomes; and longitudinal studies to understand the value of pets to healthy aging (66, 126, 127).

In their Consensus statement (124) the NIH reported the “need to recognize the heterogeneity of the older population and the complexity of the human-animal bond. … There is a need to specify the meaning of pets in everyday life and to explore the ways in which the presence of pets can affect the health and well-being of different segments of the older population” (128).

In addition to the call for more research, the development of special pet-care programs and services for and by older adults is recommended together with the implementation of protocols and guidelines for the admission of pets and visiting dogs to assisted living and institutionalized care settings (67).

### Challenges and Potential Solutions to Owning Dogs for Older Adults

Many studies have shown benefits of dog ownership for older adults. Unfortunately, many are denied this pleasure because of negative attitudes and perceived obstacles by the person themselves [e.g., the inconvenience of pet care or restrictions on freedom to travel, (129)] or their family, carers or health professionals concerned about the financial cost, the risk of zoonoses, infections or extra workload (67). Pet ownership declines with age—nearly 40% of adults in the US aged 50–67 have a pet but this declines to only 9% for those aged 68 and over (130). Many of these risks are relatively small. The risk of zoonotic infection is small other than to certain particularly vulnerable populations (67). Common concerns and barriers related to pet ownership include finances, functional capability to meet the pet's needs, restrictions imposed by their living arrangements and concerns should the pet owner fall ill or die (67, 131).

Some concerns may be relatively easily overcome with extra support from family or carers, or from external services, although there may be an associated cost. So how can older adults be supported to maintain or initiate responsible pet ownership to enjoy the companionship and health benefits dogs may bring, or to find alternative ways of interacting with dogs without the responsibility of owning a dog?

As older adults age they may become physically weaker, which may compromise their ability to interact with and adequately care for their dog (67, 127, 131). Heart and lung disease, osteoarthritis, or loss of sight may compromise their ability to adequately exercise their dog (127) or manage the motor skills necessary for attaching leads, lifting pet food or driving their dog to vet appointments or to the dog park. Older adults with dementia, still living independently, may lack the planning, organizational, and memory skills to safely care for a dog (127).

Addressing these concerns and potential barriers requires education of older adults about responsible pet ownership and welfare standards for animals kept as companions. Talking about budget, future plans and health with older adults interested in getting a pet will help them and their families and carers make good decisions (e.g., appropriate pets for their lifestyle and health status; basic discussions about exercise needs and financial resources needed to adequately care for the pet; decisions about care of the dog if the older person can no longer adequately care for their pet or they have died). Resources on how to re-home a pet safely if needed should be shared to plan for a time when they may no longer be able to meet the needs of their pet. Potential or aging owners should be made aware of existing programs that would enable them to interact with pets without having the responsibilities involved with owning them (e.g., participation in socialization programs for shelter pets, fostering shelter dogs, or keeping a dog company while friends, neighbors, or relatives are away from home working or traveling) (132).

Dog walking in particular, may be especially relevant for older adults. Maintaining physical functioning as we age is critical to maintaining independence and preventing the move from independent living to nursing homes (83). If an older person is no longer able to adequately care for a dog, they may still be able to have contact with dogs through informal dog walking groups or by walking “loaner dogs” or local shelter dogs (64). Pet visitation programs are offered in some day centers, seniors residential homes, hospitals and hospices. Some older adults may be referred for canine therapeutic programs, which support maintenance of functionality (133), orpartnered with social care providers (e.g., Meals on Wheels for their Pet Feeding programs). Increasingly, dog cafes and dog date programs are being set up to connect lonely people with dogs who enjoy human attention (134, 135).

The challenges of caring for a dog as an owner ages are compounded when older adults' dogs themselves age and require more medical attention and care. As dogs age, their declining health and strength can impact the relationship with their owner, the health benefits they can bring to the owner and the enjoyment of dog ownership. For example, sensory loss, osteoarthritis and other conditions can affect canine mobility, cognitive function and ability to locate, attend, engage and move comfortably. Walks may become less enjoyable as dogs slow down or struggle to walk on rough ground (136). Aging owners may struggle to carry their dog if it can no longer navigate stairs or steps to go outside (67).

There are clearly many challenges, but we also see exciting opportunities. As we mentioned at the beginning of this article, thinking about issues related to aging creates the opportunity for conceptually innovative interdisciplinary thinking. As we age, our companion dogs might help us to better understand and better cope with the physical challenges that we face. At the same time, our aging companion dogs create a living laboratory in which we can learn to work through challenging social and philosophical problems that we might also face with our loved ones and ourselves. How do we weight economic costs in decisions about late-life health care? How do we prioritize maximizing healthspan vs. lifespan, and how do we work through decisions that require prioritizing one over the other? What do we do with a sick pet when the effect of the treatment might be harder to bear than the disease? And how do we make the wisest choices around end-of-life/palliative care in our pets?

## Discussion

The need remains for high quality research that examines specific sub-populations of older adults. Research findings are currently mixed but we have an encouraging foundation on which to build. We know enough, and we owe it to the millions of older adults, to collectively engage and mobilize their stakeholders with the promise of science to potentially benefit many lives. The call for more, better quality HAI research has been heard before (127). There remain questions about the efficacy of Animal-Assisted Interventions and which are the most effective elements, about dosage, the time course of effects, the populations most likely to benefit and the role of lifetime pet ownership/interaction history. The National Institutes of Health/Mars Inc. Public-Private Partnership has shown the impact a concerted focus can have on a specific area of HAI research (125), not only in terms of funding, but also in guiding key stakeholders as to what to focus on and how to prioritize research topics. A similar focus is needed for older adults. New initiatives such as the Consortium on Social Isolation and Companion Animals (137) have made a good start on bringing together researchers and other stakeholders but the need remains for greater, sustained funding and research support of this area, which has so much potential to improve human health in a rapidly aging world.

## Author Contributions

SM and DP helped conceive, outline, write, and edit this manuscript. Both authors contributed to the article and approved the submitted version.

## Conflict of Interest

SM is a paid consultant, paid for by Annenberg PetSpace to lead the development of this special topic, the manuscripts of which, came from two workshops which they sponsored. DP is a paid consultant on the Research Advisory Board for the Waltham Centre for Pet Nutrition, Mars UK.
